# Antibacterial, Antioxidant, and Phytotoxic Potential of Phytosynthesized Silver Nanoparticles Using *Elaeagnus umbellata* Fruit Extract

**DOI:** 10.3390/molecules27185847

**Published:** 2022-09-09

**Authors:** Hafsa Zulfiqar, Muhammad Shoaib Amjad, Ansar Mehmood, Ghazala Mustafa, Zakia Binish, Samiullah Khan, Huma Arshad, Jarosław Proćków, José Manuel Pérez de la Lastra

**Affiliations:** 1 Department of Botany, Women University of Azad Jammu & Kashmir Bagh, Azad Kashmir 12500, Pakistan; 2 School of Geography, Earth and Environmental Sciences, University of Birmingham, Birmingham B15 2TT, UK; 3 Department of Botany, University of Poonch Rawlakot, Rawalakot 12350, Pakistan; 4 Department of Plant Sciences, Quaid-i-Azam University, Islamabad 45320, Pakistan; 5 Department of Plant Biology, Institute of Environmental Biology, Wrocław University of Environmental and Life Sciences, Kożuchowska 5b, 51-631 Wrocław, Poland; 6 Biotecnología de Macromoléculas, Instituto de Productos Naturales y Agrobiología, (IPNA-CSIC), 38206 San Cristóbal de la Laguna, Spain

**Keywords:** Ag nanoparticles, antibacterial, *Elaeagnus umbellata*, phytotoxic activity

## Abstract

Due to its eco-friendliness, cost-effectiveness, ability to be handled safely, and a wide variety of biological activities, the green plant-mediated synthesis of nanoparticles has become increasingly popular. The present work deals with the green synthesis and characterization of silver nanoparticles (AgNPs) using *Elaeagnus umbellata* (fruit) and the evaluation of its antibacterial, antioxidant, and phytotoxic activities. For the synthesis of AgNPs, fruit extract was treated with a 4 mM AgNO_3_ solution at room temperature, and a color change was observed. In UV-Visible spectroscopy, an absorption peak formation at 456 nm was the sign that AgNPs were present in the reaction solution. Scanning electron microscopy and physicochemical X-ray diffraction were used to characterize AgNPs, which revealed that they were crystalline, spherical, and had an average size of 11.94 ± 7.325 nm. The synthesized AgNPs showed excellent antibacterial activity against *Klebsiella pneumoniae* (14 mm), *Staphylococcus aureus* (13.5 mm), *Proteus mirabilis* (13 mm), and *Pseudomonas aeruginosa* (12.5 mm), as well as considerable antioxidant activity against DPPH with 69% inhibition at an IC_50_ value of 43.38 µg/mL. AgNPs also exhibited a concentration-dependent effect on rice plants. Root and shoot length were found to be positively impacted at all concentrations, i.e., 12.5 µg/mL, 25 µg/mL, 50 µg/mL, and 100 µg/mL. Among these concentrations, the 50 µg/mL concentration of AgNPs was found to be most effective. The plant biomass decreased at higher AgNP exposure levels (i.e., 100 µg/mL), whereas 50 µg/mL caused a significant increase in plant biomass as compared to the control. This study provides an eco-friendly method for the synthesis of AgNPs which can be used for their antibacterial and antioxidant activities and also as growth promoters of crop plants.

## 1. Introduction

Nanotechnology is a rapidly developing field which has greatly impacted human life in recent years. Nanoparticles are particles with sizes ranging between 1 and 100 nm. These particles possess distinctive biological and physicochemical properties due to their shape, size, composition, morphology, and surface area to volume ratio [1]. Unlike bulk materials, they have unique qualities such as good catalytic power, large surface area, thermal stability, and enhanced reactivity [2]. Because of these properties, nanoparticles have remarkable applications in the fields of medicines, manufacturing, electronics, agriculture and water treatment [3]. Various metal or metal-based oxide nanosystems have been synthesized with applications in catalytic and antibacterial fields [4,5,6,7,8,9,10,11,12,13,14]. Among different types of nanoparticles, silver nanoparticles (AgNPs) have been especially focused on due to their specific physiochemical and optoelectronic properties, which make them good fungicidal, bactericidal, anticancer, and catalytic agents [15,16]. Moreover, AgNPs are more prestigious due to their antibiotic resistance properties against multidrug-resistant bacteria. AgNPs have intense antibacterial effects against many infectious microorganisms such as *Staphylococcus aureus* and *Escherichia coli* [17].

Currently, various protocols, i.e., chemical, physical, and biological methods, are being used for the synthesis of AgNPs [18]. However, the physical and chemical methods have many drawbacks. For example, the physical methods require particular pressure and temperature conditions and are time- and money-consuming [19]. The chemical methods, although they can produce nanoparticles in suspension that can be used for functionality testing, also require the use of toxic and expensive chemicals, and the production of such products can be very hazardous to both human health and the environment [20]. 

The “green approach,” a sustainable bottom-up synthesis technique that uses biological materials such as microorganisms (bacteria, yeast, and fungi) or plant extracts to produce nanoparticles, is growing in popularity today. The aforementioned method is straightforward and environmentally friendly because it does not call for extreme pressure, pH, or temperature conditions, is economical, and does not generate toxic byproducts like some chemical processes might. As a result, the green synthesis of silver nanoparticles using plant extracts and their biological activities have been widely investigated in recent years. Alkaloids, sterols, terpenoids, tannins, saponins, flavonoids, and phenolic compounds are some of the phyto-constituents found in biological extracts used to synthesize nanoparticles. These compounds function as reductants, stabilizers, and capping adaptors during the synthesis stage [21]. The properties of AgNPs depend on their shape, size, and morphology. Biosynthesized AgNPs exhibit different shapes, sizes, and morphologies. Different experimental conditions such as pH, temperature, reducing agents, nature, and the adsorption of capping agents and the kinetics of interaction between metal salts strongly influence these properties. Researchers have used various plants or plant parts for the synthesis of various nanoparticles such as silver [22], gold [23], iron oxide [24], and zirconia [25].

*Elaeagnus umbellata* (Thumb), which belongs to the Elaeagnaceae family, is a wild shrub deciduous species that exists at an altitude of 4500 to 6000 feet above sea level in the different regions of the Himalayas in Pakistan [26,27]. Its berries contain significant medicinal properties [28]. The fruit of *E. umbellata* shows 17 times more antioxidant activity (lycopene) than *Lycopersicum esculentum* [29]. It works against various diseases such as fever and many types of cancers [27]. The fruit extract has been reported to be effective against bacterial infections and other secondary complications associated with type 2 diabetes [30,31]. *E. umbellata*’s flowers and seeds are very useful as they are used as a tonic to cure coughs. The seeds are also used to extract oil which is used in the treatment of pulmonary infections [32]. The *E. umbellata* essential oil possesses antioxidant anticholinesterase and antidiabetic potential [33]. The phytochemical structures of these plants contain saponins and steroids which make them beneficial to nanoparticle synthesis [34]. The plant is also known for its antimicrobial properties and is used for the treatment of infections [34].

To date, no research has been conducted to examine the synthesis of AgNPs from *E. umbellata* fruit extract and their multidirectional functions as antibacterial, antioxidant, and phytotoxic nanoparticles, despite the fact that AgNPs made from plant extract have demonstrated antibacterial and antioxidant activity. Therefore, the main objective of this work was to synthesize and characterize AgNPs from *E. umbellata* fruit extract. The second objective was to examine the antibacterial, antioxidant, and phytotoxic properties of biosynthesized AgNPs.

## 2. Materials and Methods

### 2.1. Collection of Plant Material

*Elaeagnus umbellata* (fruit) was collected from the nearby areas of Rawalakot, Abbaspur, and Hajira. The plant was identified by a taxonomist at the Department of Botany, Women University of Azad Jammu & Kashmir, and with the help of the flora of Pakistan. A voucher specimen was deposited in the herbarium at the Women University of Azad Jammu & Kashmir.

### 2.2. Preparation of Plant Extract (Fruit)

Firstly, the fruits of the plant were washed many times with normal tap water and then with distilled water to remove all the dust particles, and they were dried in the shade to remove the residual moisture. After 25 days, the dried fruits were ground to a powder form and stored in an airtight jar for further use. For the preparation of the plant extract, 10 g of powder was dissolved in 100 mL of distilled water and left for 3 days. It was then filtered using Whatman No.1 filter paper, and the filtrate was saved in a test tube for the synthesis of AgNPs.

### 2.3. Synthesis of AgNPs

The AgNPs were synthesized by using the green synthesis approach. For the synthesis, AgNO_3_ (80 mL; 1.0 mM) solution was added to 20 mL of plant filtrate in a flask. This mixture was kept for 30 min at 45 °C in a water bath by adjusting the pH to 9, as higher pH values have been shown to result in smaller-sized nanoparticles [35]. The resulting solution was kept in the dark, and we waited for a color change to take place. After a certain time, the solution’s color changed from transparent to yellow and then to dark brown, which indicated the synthesis of AgNPs. The solution thus obtained was poured into falcon tubes and centrifuged at 10,000 rpm for 6 min. The process of re-dispersion and re-centrifugation was repeated thrice to remove any unwanted material.

### 2.4. Characterization of AgNPs

The synthesized AgNPs were characterized by using the following techniques: 

#### 2.4.1. UV-Visible Spectroscopy

UV-Visible spectroscopy is a broadly used tool to characterize nanoparticles. The aqueous solution of synthesized AgNPs was prepared using distilled water (DW). The solution was sonicated for 5–10 min, and absorbance was measured between 300 and 700 nm using the Cary E 5000 spectrophotometer (Agilent Technologies Inc., Santa Clara, CA, USA), which indicated the synthesis of AgNPs. 

#### 2.4.2. X-ray Diffraction (XRD)

X-ray diffraction spectroscopy is a technique used to study the morphology, size, and structure of nanoparticles. For the X-ray diffraction analysis, a finely ground or powdered sample of silver nanoparticles was prepared, which was then purified via centrifugation at 14,000 rpm for 5 min. After the centrifugation, the pellet was dried at room temperature and then examined by using “Bruker-D8-advance XRD” (Bruker AXS GmbH, Karlsruhe, Germany) in the range of 5–500 at a 2θ angle. The size of the particles was calculated using the formula given by Debye-Scherrer, and synthesized silver nanoparticles were characterized by using the following techniques: 

D = k λ/βcosθ; 

K = shape factor;

λ = wavelength of X-ray; 

β = full width in radian in half maximum;

θ = Bragg’s angle.

#### 2.4.3. Scanning Electron Microscopy (SEM)

The surface morphology or structural analysis of the synthesized AgNPs was inspected using SEM, using the model “Jeol JSM-6490A” (JEOL Ltd., Tokyo, Japan) analytical scanning electron microscope, which was operated at 5 Kv at a magnification × 10 Kv, from the Pakistan Institute of Engineering and Applied Sciences (PIEAS), Islamabad. The sample for SEM analysis was prepared by placing AgNPs in water on a carbon-coated copper grid and drying them completely using blotting paper, followed by placing them under a mercury lamp for 5 min. Images of the sample were taken, and a size distribution histogram of the AgNPs was made using ImageJ software.

#### 2.4.4. Energy-Dispersive X-ray (EDX)

The presence of metallic silver ions or elemental analysis was examined by using an EDX detector (Bruker Quantax, Bruker Corporation, Billerica, MA, USA). The synthesized AgNPs were poured on the carbon film and dried. The spectrum obtained from the sample was then analyzed by using a semiconductor for the detection of X-rays together with electronic processing.

#### 2.4.5. Fourier Transform Infrared (FTIR) Spectroscopy

FTIR spectroscopy(FTIR-4100 spectrometer, Jasco, Japan) is a technique that is used to verify the functional groups and adsorbed chemicals that are helpful in the capping and stabilization of nanoparticles [36,37]. An Elmer FTIR spectrophotometer was used for the FTIR measurement of the sample using a standard KBr pellet technique. The powder silver nanoparticles were scanned in the range of 400–4000 cm^−1^ using a spectrometer operating at a resolution of 4 cm^−1^. 

### 2.5. Antibacterial Activity

The antimicrobial potential of the synthesized AgNPs was studied against clinically isolated Gram-positive (*Staphylococcus aureus*) and Gram-negative (*Klepsiella pneumonia*, *Proteus mirabilis*, and *Pseudomonas aeruginosa*) bacteria by using the disc diffusion method [38]. Nutrient agar medium was prepared by adding 28 g of agar in 1000 mL of distilled water for bacterial cultivation. The agar medium and the other necessary items such as Petri plates, filter paper discs, loopers, test tubes, spatulas, and forceps were autoclaved for 15 min at 121 °C. Afterwards, all of the autoclaved items were kept in the incubator at 37 °C to dry. The agar medium was poured on a plate and was allowed to solidify. Bacterial strains were inoculated on the nutrient agar plates. AgNPs were dissolved in distilled water to prepare solutions of 30, 60, and 100 mg/L concentrations. Filter paper discs were dipped in each solution of AgNPs, allowing the excess solvent to evaporate. The discs were placed around the plates with the standard ampicillin disc, which was used as a positive control in the middle of the plate. The bacterial strains were incubated at 37 °C for 24 h. Untainted Solvent and distilled water were used as negative controls. Microbial growth was determined by measuring the diameter of the zone of inhibition against each bacterium. From this test, the controlled bacterial activity was subtracted, and the attained results were plotted. The percentage inhibition zone was determined by using the given formula (Equation (1)):

(1) $$\mathrm{Percentage}\;\mathrm{inhbitaion}\;\mathrm{zone}=\frac{\mathrm{zone}\;\mathrm{of}\;\mathrm{inhibition}\;\mathrm{of}\;\mathrm{sample}}{\mathrm{zone}\;\mathrm{of}\;\mathrm{inhibition}\;\mathrm{of}\;\mathrm{standard}}\times 100$$

### 2.6. Antioxidant Activity

The antioxidant potential of the synthesized AgNPs was determined by a free radical scavenging assay using 1.1-diphenyl-2-picrylhdrazyl (DPPH) [39]. DPPH solution was prepared by adding 12.5 mg of DPPH powder to 50 mL of methanol. The plant sample solution was also prepared in methanol (1 mg/mL). The serial dilution of the solution was also prepared with different concentrations (100, 50, 25, and 12.5 µg/mL). Further, 0.1 mL of each dilution was mixed with 3.0 mL of DPPH in a test tube and kept in the incubator for half an hour at 37 °C. Ascorbic acid was used as the standard. A UV-visible spectrophotometer was used to determine the absorbance against the standard at 517 nm. All the test tubes were examined three times for absorbance. The control sample was also prepared by using 2 mL of DPPH solution + 1 mL of methanol. The result percentage inhibition was calculated by using the given formula (Equation (2)):

(2) $$\%\;\mathrm{inhbition}\;\mathrm{of}\;\mathrm{DPPH}=\frac{\mathrm{Absorbance}\;\mathrm{of}\;\mathrm{control}\;-\;\mathrm{absorbance}\;\mathrm{of}\;\mathrm{sample}}{\mathrm{Absorbance}\;\mathrm{of}\;\mathrm{control}}\times 100$$

### 2.7. Phytotoxicity Assessment of AgNPs

The phytotoxic potential of the synthesized nanoparticles was assessed on rice seedlings (Oryza sativa var. IR-6) at four different concentrations, i.e., 12.5 µg/mL, 25 µg/mL, 50 µg/mL, and 100 µg/mL. Healthy seeds of *Oryza sativa* (IR-6) were collected from the Crop Science Institute, NARC (National Agriculture Research Center) Islamabad Pakistan. Seeds were surface sterilized with 3% sodium hypocrite solution and were sown in the sand. The pots were then shifted into a growth chamber with 47% humidity, a 16 ± 8 h light and dark period, and a constant temperature of 25 °C. Two-week-old plants were treated with nanoparticles, and the morphological parameters of fresh plant weight, shoot weight, and root weight were observed for three consecutive days for each treatment. The root and shoot lengths of the rice seedlings were measured using a measuring scale in cm on the 15th, 16th, and 17th day of sowing. The fresh weight of the plant was measured using an electrical weighing balance (mg). All the experiments were performed in triplicate.

## 3. Results and Discussion

### 3.1. Synthesis of AgNPs 

**Figure 1:**
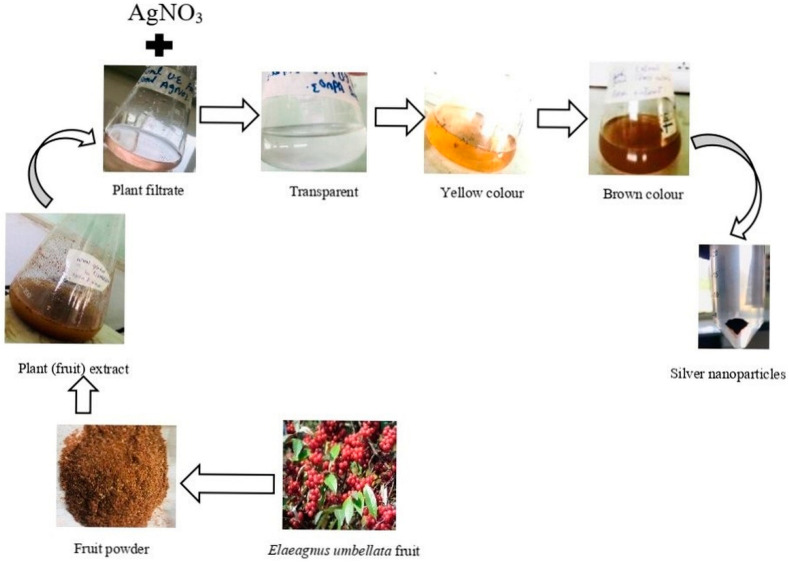
Synthesis of silver nanoparticles using *Elaeagnus umbellata fruit* extract.

In the present study, fruits of *Elaeagnus umbellata* were taken from Rawalakot, district Poonch AJK for the green synthesis of AgNPs. The *E. umbellata* plant was selected due to its medicinal properties. When plant extract was treated with silver nitrate solution, the color of the reaction solution started to change from transparent to yellow, finally developing into a brown color (Figure 1). This color change is the first indication of silver ion (Ag+) reduction to silver nanoparticles [40]. The Surface Plasmon resonance of AgNPs is responsible for the change in the color of the reaction solution [41]. Previous studies also supported this fact that the color of the reaction solution of AgNO_3_ and plant extract changes with time [42,43].

### 3.2. Characterization of AgNPs

#### 3.2.1. UV-Visible Spectroscopy

**Figure 2:**
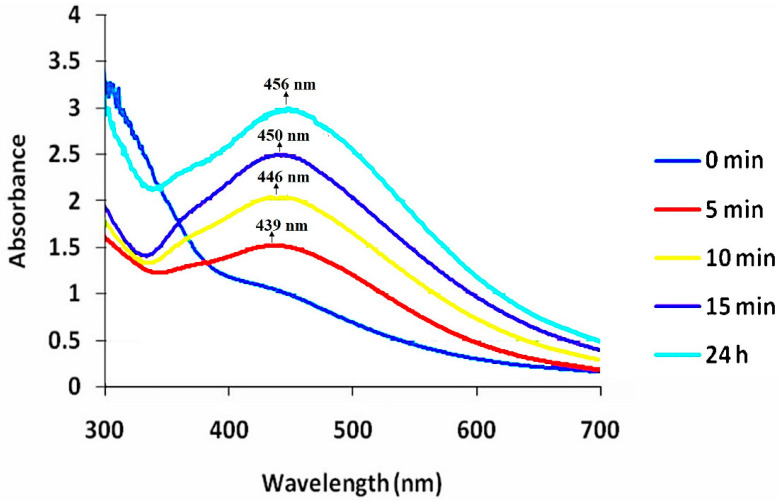
UV-Vis absorption spectra of silver nanoparticles at different time intervals synthesized from *Elaeagnus umbellata* fruit extract.

UV-Vis spectroscopy is a widely used technique to confirm the synthesis formation of silver nanoparticles (AgNPs) in the reaction solution of AgNO_3_ and plant extract. The reaction solution was scanned between λmax 300 and 700 nm at different time intervals. There was no absorption peak. Different characterizations peaks usually occur between 410 and 480 nm for AgNPs in this solution. Various shapes and sizes of silver nanoparticles are attributed to different wavelengths. The synthesized AgNPs were observed by recording a UV-visible spectrum from 300 to 700 nm. At zero time, no peak was formed, but with the passage of time from 5 min, a formation of an absorption peak was recorded. The intensity of the absorption peak was increased with the passage of time from 5 min to 24 h (Figure 2). Moreover, the shifting of the absorbance peak of AgNPs was also recorded with time. Initially, the peaks were recorded at 10, 15 min, and 24 h as absorbance increased, and peaks were formed. The first peak appeared after 5 min at 439 nm (after 5 min), which was shifted to 446 nm, (after 10 min) at 446 nm, 450 nm (after 15 min) and finally at 456 nm (after 24 h). This shifting of the absorption peak was linked to changes in particles (both increases and reductions). The blue shift indicated a decrease in size, whereas the red shift indicated an increase in size [44]. This seemed to occur at 450 nm and 456 nm, respectively (Figure 2). The result of UV-Vis spectroscopy initially showed the increased intensity of the Plasmon band and later showed lower intensity due to the smaller size of the particles. Peaks were confirmed by the earlier studies by Shah et al. [45].

#### 3.2.2. X-ray Diffraction (XRD)

**Figure 3:**
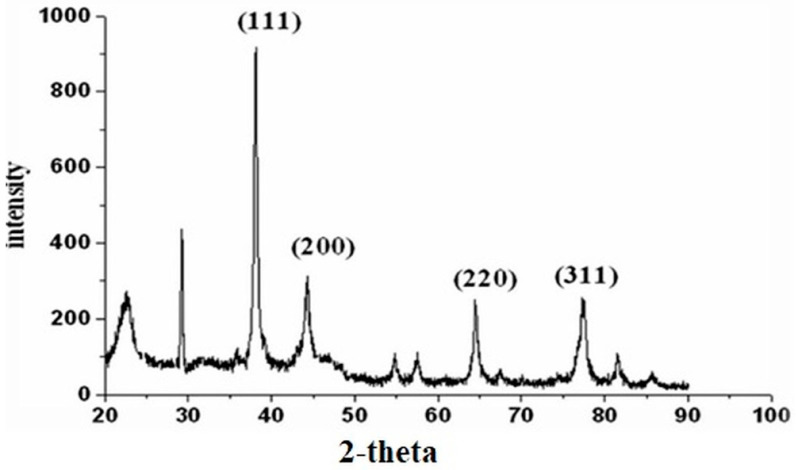
X-ray diffraction pattern of synthesized silver nanoparticles of *Elaeagnus umbellata* fruit extract.

The results of XRD showed diffraction patterns of synthesized AgNPs at 2Ѳ peak values around 38.17°, 44.31°, 64.44°, 77.34°, and 81.33°, which can be indexed to the face-centered-cubic crystalline pattern (111), (200), (220), (311), and (222), an index of silver [46]. This shows that *E. umbellata* fruit-mediated silver nanoparticles were crystalline in structure. (Table 1; Figure 3). In addition to Bragg’s peaks, the recorded XRD pattern revealed other unassigned peaks. These peaks could be the result of bioorganic or metallo-proteins in the supernatant that are in charge of stabilizing nanoparticles [47].

The intensity of Bragg’s reflection at 38.17° was a good signal that fruit-mediated silver nanoparticles were cubic. The lattice constants ‘a’ were calculated as 4.106 nm, which agreed well. The size of the AgNPs was calculated by using the FWHM value in the Debye-Scherrer formula which confirmed that the nanoparticles were in the nanometer range of Ag particles. The average size of *E. sumbellata* fruit-mediated silver nanoparticles was 13.45 ± 7.69 nm. The size of *E. umbellata* fruit AgNPs ranged from 7.56 to 24.43 nm with an average size of 13.45 nm (Table 1; Figure 3).

#### 3.2.3. Scanning Electron Microscope (SEM)

**Figure 4:**
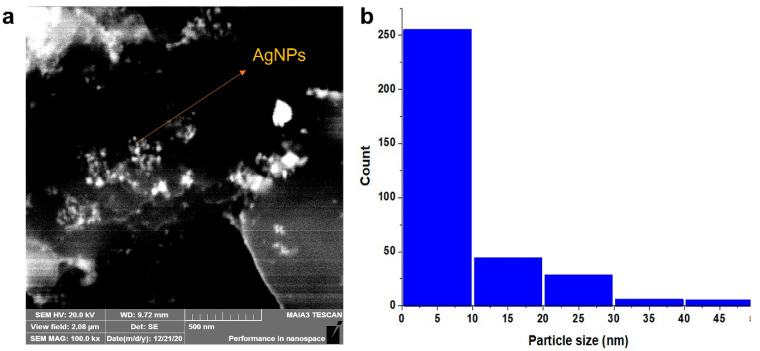
SEM analysis of AgNPs, (**a**) SEM, (**b**) particle size distribution.

SEM images of AgNPs synthesized from the plant extract of *E. umbellata* were taken to observe the size and morphology of the prepared nanoparticles (Figure 4). The results indicated the nanoparticles were spherical with an average size of 11.94 ± 7.325 nm.

#### 3.2.4. Energy-Dispersive X-ray (EDX)

**Figure 5:**
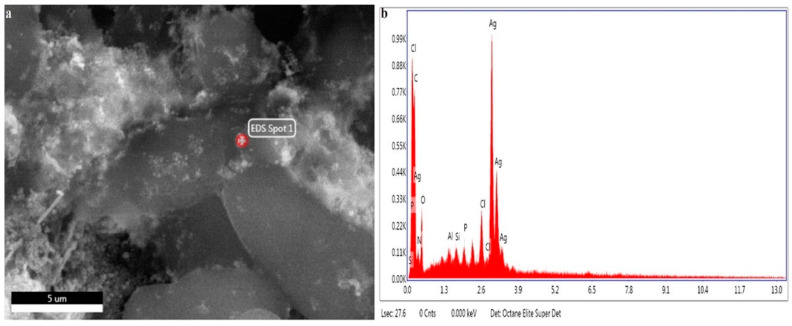
EDX analysis of AgNPs, (**a**) EDX micrograph, (**b**) EDX spectrum of AGNPs synthesized from *Elaeagnus umbellata* fruit extract.

The presence of metallic silver ions was confirmed by the EDX (Figure 5). The EDX spectrum revealed a strong absorption peak of metallic silver ions in the series of 2.5–3.5 keV, while silver nanocrystals showed absorption peaks in the series of 2.5–3.7 keV. The peaks of silver nanoparticles were at 2.7, 2.9, and 3.2 keV, which showed the presence of AgNPs in the solution (Figure 5).

Some weak peaks were also seen for chlorine at 2.5 keV, silicon at 1.4 keV, phosphorous at 1.6 keV, and aluminum at 1.3 keV, but they were due to biomolecules of bacteria responsible for the silver nanoparticles’ synthesis [48]. The result obtained from the EDX spectrum was used to define the weight percentage of silver by using quantitative analysis via the ZAF method. The percentage weight of silver nanoparticles was 67.96%, and its atomic weight was 21.88% (Table 2). Our findings were in line with some other researchers who reported the absorption peaks of the presence of the silver ions by utilizing an Energy-Dispersive X-ray detector [49].

#### 3.2.5. Fourier Transform Infrared Spectroscopy (FTIR)

**Figure 6:**
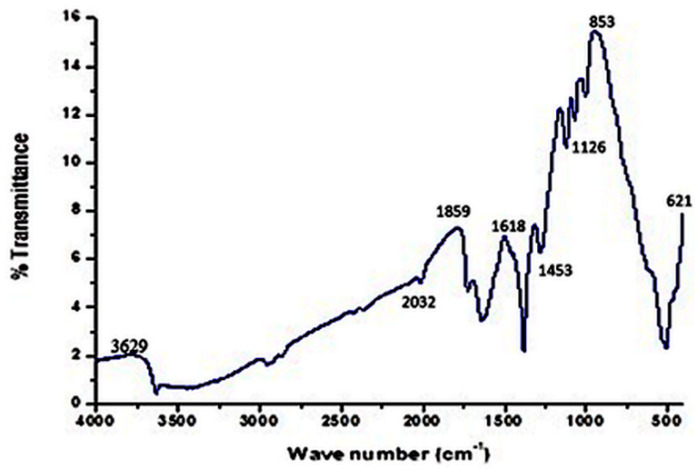
FTIR analysis of AgNPs synthesized from *Elaeagnus umbellata* fruit extract.

Fourier Transform Infrared Spectroscopy is an essential technique for molecular fingerprinting used to detect the functional group of plant secondary metabolites that act as capping or reducing agents in the synthesis of silver nanoparticles and to obtain an infrared spectrum of absorption of a solid, liquid, or gas [50]. It simultaneously collects high-resolution spectral data over a wide spectral range. Biomolecules that are associated with silver nanoparticles are detected via FTIR. These biomolecules are responsible for the reduction process from Ag^+^ to AgNPs [39,51]. The peaks of *E. umbellata* nanoparticles were observed in a range between 400 and 4000 cm^−1^. The FTIR results are given in Figure 6.

The strongest and the broadest band appeared at 3629 cm^−1^, which showed O-H linkages of phenol and alcohol bonds. At 2032, 1859, 1723, 1618, and 853 cm^−1^, strong bands of amides, alkenes, and aldehydes were shown. Medium bands appeared at 1453 cm^−1^ and 1126 cm^−1^, which showed alkane and alkoxy. Weak bands were detected at 621 cm^−1^ which were halo compounds. The Figure 6 showed a broad IR transmission band at 3629 cm^−1^. At 2032, 1859, 1723, 1618, and 853 cm^−1^, loud, noticeable bands appeared. The Fourier Transform Infra-Red Spectroscopy results of *E. umbellata* fruit AgNPs showed peaks that appeared at 3629 cm^−1^, generally expressive of alcoholic and phenolic groups capped on biogenic nanosilver powder (Table 3). The presence of aldehydes, amides, alkenes, alcohols, and halo compounds was confirmed via FTIR, which played vital roles as reducing and capping agents of AgNPs’ synthesis. These functional groups were also responsible for the stabilization of particles. Similar results from FTIR were reported earlier and hence confirmed [52].

### 3.3. Antibacterial Activity

**Figure 7:**
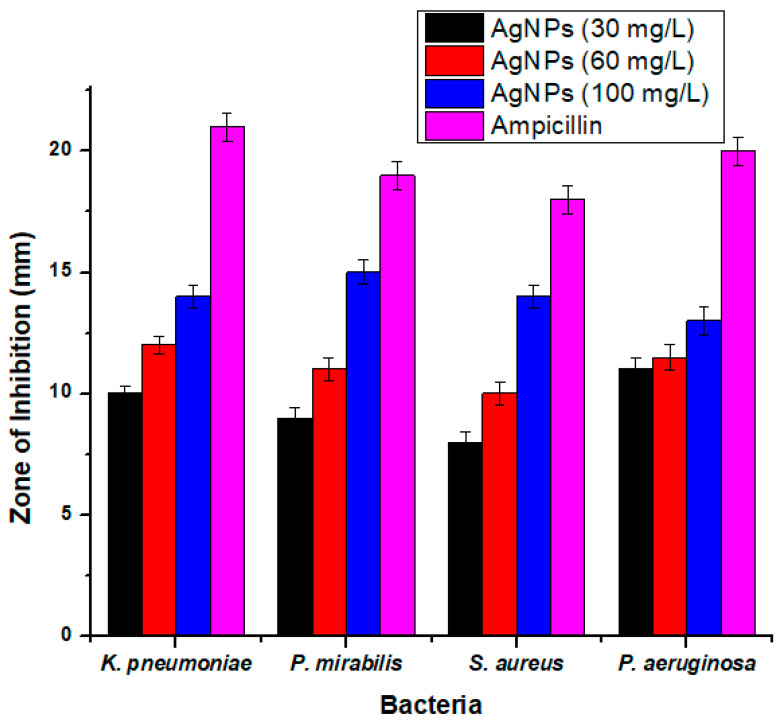
Antibacterial activity of AgNPS synthesized from *Elaeagnus umbellata* fruit extract.

The antibacterial activity of synthesized AgNPs was assessed for Gram-positive (*S. aureus*) and Gram-negative (*K. pneumonia*, *P. mirabilis*, and *P. aeruginosa*) bacteria by using the disc diffusion method. The dose-dependent antibacterial activity of AgNPs was found against all the tested bacteria. The AgNPs showed high inhibitory activity of 15 ± 0.50 mm against S. aureus, which is a Gram-positive bacterium, followed by 14 ± 0.45 mm against *K. pneumoniae* at 100 mg/L, 14 ± 0.49 mm against *P. mirabilis*, and 13 ± 0.60 mm against *P. aeruginosa* at a concentration of 100 mg/L (12 ± 0.35 mm) (Figure 7). It was also observed that the antibacterial activity of AgNPs increased with an increase in the concentration of AgNPs from 30 to 100 mg/L. 

It has been described that the antibacterial activity that was shown by the silver ion was due to the positive charge on silver and the negative charge on the cell membrane of microorganisms. Electrostatically, a positive charge exerts a pull-on negative charge [53]. Chen at el. showed that silver joins with bacteria due to their inimitable size and vital surface area [54].

Stoimenov et al. [55] confirmed that AgNPs bind with the DNA of bacteria and are then attached to ribosomes of bacteria; there, they prevent DNA duplication and cause bacterial death. The most typical aspect in an antibacterial study is the interaction of a positive charge on AgNPs and a negative charge on bacteria which may kill the bacteria. The details of this are shown below.

### 3.4. Antioxidant Activity

**Figure 8:**
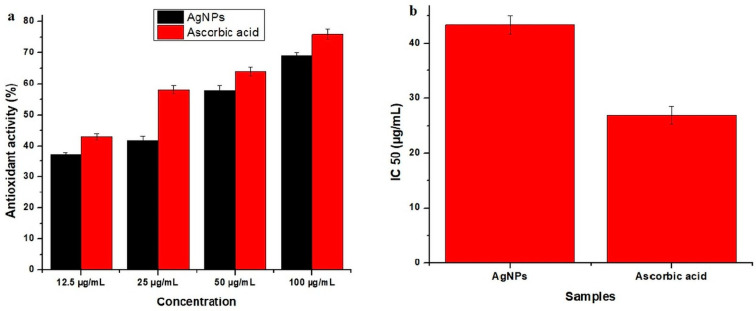
DPPH scavenging activity of AgNPs, (**a**)% antioxidant activity at different concentrations, (**b**) IC_50_ value at different concentrations.

The most common method which is used to determine the antioxidant activity of plants is DPPH for free radicles. DPPH is a kind of free radical that authenticates the inspection of primary radical scavenging activity. In the current study, ascorbic acid was used as a control, and its absorbance was about 0.80. The AgNPs synthesized from *E. umbellata* fruit extract showed good antioxidant potential of 69% at 100 µg/mL and moderate potential of 57.8%, 41.8%, and 37.30% at 50 µg/mL, 25 µg/mL, and 12.5 µg/mL, respectively (Figure 8). 

Antioxidant activity was examined because most of the sicknesses in humans are due to free radicals. Free radicals cause many diseases in the human body. Free radicals that are produced in our body are examined by antioxidant monitoring compounds. There are many kinds of antioxidants that are not good for human health, but they are swapped for natural ones [56]. There are many kinds of human diseases, for example, inflammatory diseases, cardiovascular diseases, and cancer, in which antioxidants reduce the oxidative stress in cells and make it useful in many aspects [57]. To avoid artificial antioxidants, more cheap, bioactive, and safe antioxidants have been explored from plants [58]. Many studies have confirmed the antioxidant activity of medicinal plants. AgNPs showed good antioxidant activity, as they reduce the oxidative stress in cells, which is beneficial for many ailments such as cancer, pulmonary infection, and cardiovascular and inflammatory infection. These results have been studied, compared, and confirmed by earlier researchers [59].

### 3.5. Phytotoxicity Assessment of Synthesized AgNPs

**Figure 9:**
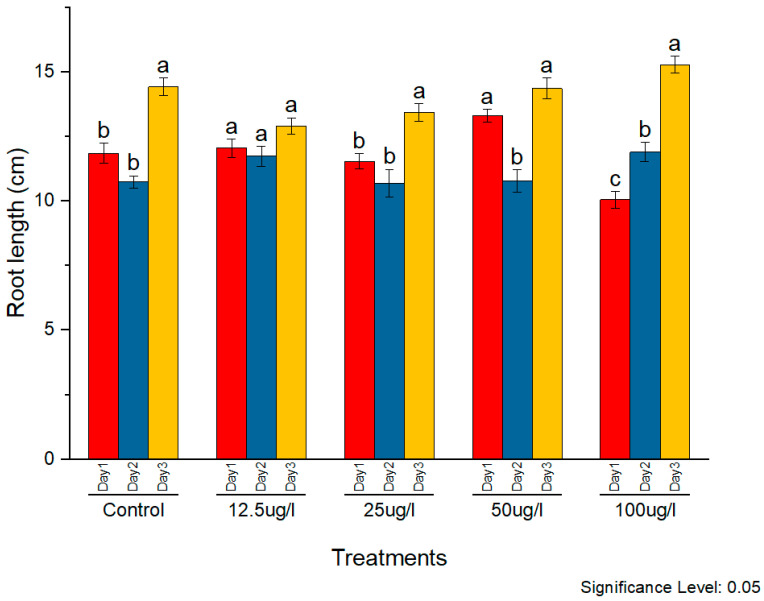
Phytotoxic effects of AgNPs on plant root, a and b indicates two significance sub groups based on Latin Square Design (LSD) value.

**Figure 10:**
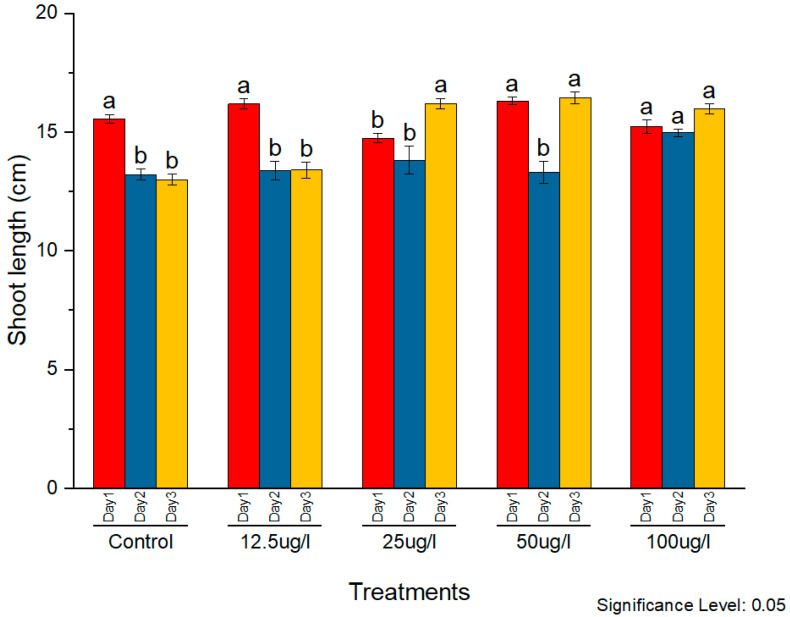
Phytotoxic effects of AgNPs on plant shoot, a and b indicates two significance sub groups based on Latin Square Design (LSD) value.

**Figure 11:**
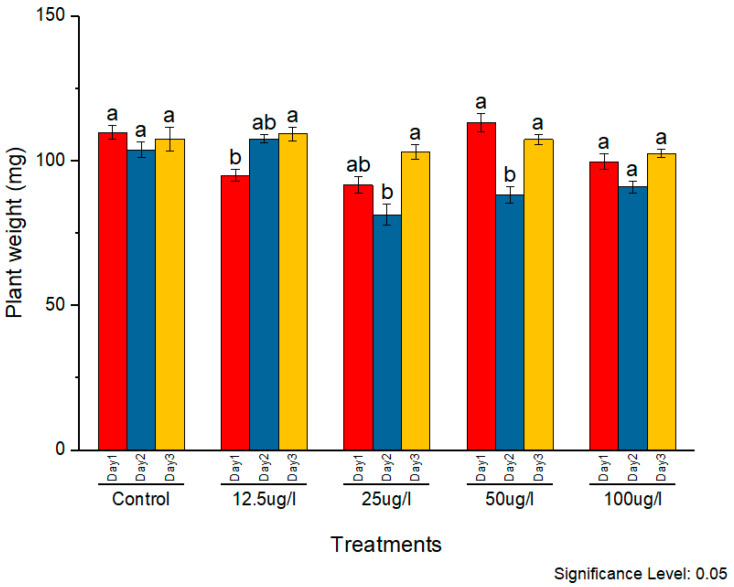
Phytotoxic effects of AgNPs on plant weight, a and b indicates two significance sub groups based on Latin Square Design (LSD) value.

The phytotoxicity of the synthesized AgNPs was assessed on rice seedlings. Silver nanoparticles in the concentrations of 12.5, 25, 50, and 100 µg/mL were applied to two-week-old seedlings. The morphological parameters of plant weight, root length, and shoot length were observed and compared with the control. The subsequent effects on plant morphology were observed for three consecutive days. All concentrations caused noticeable positive effects on all morphological parameters with constant external conditions (Figure 9, Figure 10 and Figure 11, respectively).

Silver nanoparticles were found to positively impact root length at all concentrations as compared to the control. A maximum increase was observed at 50 µg/mL, although other concentrations also exhibited a significant increase in root length as well.

A similar effect was observed on the shoot length as well. On day 1, plants treated with 12.5 µg/mL showed the maximum shoot length, whereas later on, 50 µg/mL was found to cause the maximum increase. However, this increase in shoot length was observed at other concentrations as well on all three days.

In the case of plant weight, 50 µg/mL was found to be more effective as compared to other concentrations. It caused a significant increment in plant weight on all three days. Initially, 12.5, 25, and 100 µg/mL caused a decrease in plant weight as compared to the control. On the day 2, 100 µg/mL caused the maximum decrease. On day 3, increased plant biomass was observed at 50 µg/mL, whereas other concentrations caused a decreased plant biomass as compared to the control.

Thus, it can be concluded that the nanoparticle application caused significant changes in the morphology of the plants. These changes were found to be more pronounced in the root as it is the first organ that encounters the nanoparticles. This increase was found to be dose-dependent. The positive correlation between the phytotoxicity and the concentration of AgNPs during exposure has been reported in several studies. The concentration of AgNPs only above the threshold level causes negative effects. In this study, the concentration above 50 µg/mL exhibited phytotoxic effects, whereas lower concentrations were found to be beneficial for plant growth. Mirzajani et al. (2012) reported phytotoxic effects of AgNPs on the cell wall, cell morphology, and other structural features. They observed enhanced root length at a 30 µg/mL concentration, while 60 µg/mL restricted root growth [60]. Positive effects of a low concentration of AgNPs on seedling development were also reported in Pisum sativum [61]. Higher concentrations of AgNPs were shown to decrease seed germination and subsequent seedling growth in jasmine rice [62]. AgNPs with moderate concentrations improved the seedling growth, while higher concentrations reduced the plant growth [63]. Similar results regarding the toxicity of AgNPs on plant morphology have been reported in various other plant species as well, including Arabidopsis, Brassica nigra, and wheat [64,65,66].

## 4. Conclusions

Nanoparticles are synthesized using different methods, including physical, biological, and chemical techniques. Biologically synthesized nanoparticles cause positive impacts on plants compared to chemically synthesized nanoparticles. In the present study, a green synthesis method utilizing *E. umbellata* fruit extract was used for the synthesis of AgNPs. The synthesized nanoparticles were characterized using SEM, EDX, XRD, and FTIR analysis. The obtained AgNPs were predominantly spherical in shape, crystalline in nature, and had an average size of 11.94 ± 7.325 nm. The EDX spectrum indicated the signature peak for silver. The FTIR analysis revealed the presence of major functional groups of important bioactive constituents such as terpenoids, flavonoids, alkane, alkene, phenols, amides, and alcohols on the surface of AgNPs. These AgNPs showed pronounced antibacterial and antioxidant activity. The nanoparticles were also found to improve plant growth up to a 50 µg/mL concentration in a dose-dependent manner. This study provides an eco-friendly, economical, and cost-effective method for the synthesis of AgNPs, which could have potential applications in the field of biomedicine, agriculture, and water treatment. However, the commercial application of AgNPs require high-yielding methods for which this synthesis method of AgNPs still requires further refinement.

## Figures and Tables

**Table 1:** Measurement the size of biosynthesized silver nanoparticles with *Elaeagnus umbellata* (fruit extract) by using Debye–Scherrer equation.

Peaks	2Ѳ	Height	FWHM	D-Spacing	Relative Intensity Rel. Int	Particle Size
111	38.17	148.65	0.386	2.376	100.11	13.10
200	42	40.87	0.623	2.053	28.34	7.56
220	65	61.76	0.204	1.048	41.12	24.43
311	78	52.69	0.644	1.245	35.16	8.74

**Table 2:** Elemental analysis of AgNPs synthesized from *Elaeagnus umbellata* fruit extract.

Element	Weight%	Atomic%	Net Int.	Error%	Kratio	Z	R	A	F
C K	29.49	48.76	259.02	8.57	0.1294	1.1354	0.9068	0.3863	1
N K	10.87	15.41	41.94	14.51	0.0153	1.1118	0.9194	0.1265	1
O K	20.43	25.36	116.08	12.18	0.0242	1.0909	0.9305	0.1085	1
AlK	1.39	1.03	51.69	14.57	0.0075	0.9772	0.9763	0.5466	1.0094
SiK	1.28	0.91	56.73	15.05	0.0086	0.9997	0.9839	0.6644	1.0146
P K	1.25	0.8	52.77	13.47	0.0093	0.9612	0.9912	0.7593	1.0227
ClK	3.31	1.86	146.92	7.51	0.029	0.9341	1.0048	0.8936	1.0466
AgL	67.96	21.88	658.09	2.12	0.2706	0.7525	1.201	1.1087	1.0144

**Table 3:** FTIR peaks and possible functional groups of *Elaeagnus umbellata* nanoparticles.

*Elaeagnus umbellata* Fruit Mediated AgNPs	Stretching	Bond Type	Possible Compounds
FTIR Frequency (cm^−1^)	Intensity		Functional group
621	W	C-X	Halo compound
853	W	C=C	Alkene
1126	M	C-O	Alkoxy
1453	M	C-C	Alkane
1618	S	C=C	Alkene
1723	S	C=O	Aldehydes
1859	S	C=O-NR_2_	Amides
2032	S	C≡C	Alkynes
3629	Br	O-H	Alcohols

Key: M = medium, W = weak, S = strong, and Br = broad.

## Data Availability

The data presented in this study are available on request from the corresponding author.

## Sample Availability

Samples of the compounds are not available from the authors.
